# Current status of the multinational Arabidopsis community

**DOI:** 10.1002/pld3.248

**Published:** 2020-08-02

**Authors:** Geraint Parry, Nicholas J. Provart, Siobhan M. Brady, Baris Uzilday, The Multinational Arabidopsis Steering Committee, Keith Adams, Wagner Araújo, Sébastien Aubourg, Sacha Baginsky, Erica Bakker, Katja Bärenfaller, Jacqui Batley, Mike Beale, Mark Beilstein, Youssef Belkhadir, Gregor Mendel, Tanya Berardini, Joy Bergelson, Francisca Blanco‐Herrera, Siobhan Brady, Hans‐Peter Braun, Steve Briggs, Lynette Brownfield, Maura Cardarelli, Marcos Castellanos‐Uribe, Gloria Coruzzi, Maheshi Dassanayake, Geert De Jaeger, Brian Dilkes, Colleen Doherty, Joe Ecker, Pat Edger, David Edwards, Farid El Kasmi, Maria Eriksson, Moises Exposito‐Alonso, Pascal Falter‐Braun, Alisdair Fernie, Myriam Ferro, Oliver Fiehn, Joanna Friesner, Katie Greenham, Yalong Guo, Thorsten Hamann, Angela Hancock, Marie‐Theres Hauser, Joshua Heazlewood, Cheng‐Hsun Ho, Hanna Hõrak, Eva Huala, Inhwan Hwang, Satoshi Iuchi, Pankaj Jaiswal, Liina Jakobson, Yunhe Jiang, Yuling Jiao, Alexandra Jones, Yasuhiro Kadota, Jitendra Khurana, Dan Kliebenstein, Emma Knee, Masatomo Kobayashi, Marcus Koch, Gabriel Krouk, Tony Larson, Rob Last, Loïc Lepiniec, Song Li, Claire Lurin, Martin Lysak, Steven Maere, Robert Malinowski, Florian Maumus, Sean May, Klaus Mayer, David Mendoza‐Cozatl, Isabel Mendoza‐Poudereux, José Luis Micol, Harvey Millar, Hans‐Peter Mock, Karolina Mukhtar, Shahid Mukhtar, Monika Murcha, Hirofumi Nakagami, Yasukazu Nakamura, Luke Nicolov, Basil Nikolau, Moritz Nowack, Adriano Nunes‐Nesi, Michael Palmgren, Geraint Parry, Nicola Patron, Scott Peck, Ullas Pedmale, Catherine Perrot‐Rechenmann, Roland Pieruschka, José Pío‐Beltrán, J. Chris Pires, Nicholas Provart, Loïc Rajjou, Leonore Reiser, Sue Rhee, Stamatis Rigas, Norbert Rolland, Andres Romanowski, Sigal Savaldi‐Goldstein, Robert Schmitz, Waltraud Schulze, Motoaki Seki, Kentaro K. Shimizu, Keith Slotkin, Ian Small, David Somers, Rosangela Sozzani, Charles Spillane, Ramamurthy Srinivasan, Nicolas Taylor, Marcela‐Karey Tello‐Ruiz, Jay Thelen, Takayuki Tohge, Christopher Town, Tetsuro Toyoda, Baris Uzilday, Justin Walley, Doreen Ware, Wolfram Weckwerth, Julian Whitelegge, Stefanie Wienkoop, Clay Wright, Michael Wrzaczek, Misako Yamazaki, Marcelo Yanovsky, Viktor Žárský, Xuehua Zhong, Yves Van De Peer, Klaas Van Wijk, Philipp Von Gillhaussen

**Affiliations:** ^1^ School of Biosciences Cardiff University Cardiff United Kingdom; ^2^ Department of Cell and System Biology/Centre for the Analysis of Genome Evolution and Function University of Toronto Toronto Canada; ^3^ Department of Plant Biology and Genome Center University of California Davis USA; ^4^ Department of Biology Faculty of Science Ege University Izmir Turkey

**Keywords:** *Arabidopsis thaliana*, collaboration, Research Network, roadmap

## Abstract

The multinational Arabidopsis research community is highly collaborative and over the past thirty years these activities have been documented by the Multinational Arabidopsis Steering Committee (MASC). Here, we (a) highlight recent research advances made with the reference plant *Arabidopsis thaliana*; (b) provide summaries from recent reports submitted by MASC subcommittees, projects and resources associated with MASC and from MASC country representatives; and (c) initiate a call for ideas and foci for the “fourth decadal roadmap,” which will advise and coordinate the global activities of the Arabidopsis research community.

The multinational Arabidopsis research community is highly collaborative. This has been demonstrated through global efforts to publish the Arabidopsis genome sequence in 2000; the long‐term support for the three international stock centres in the United States, United Kingdom and Japan; and in the development of community‐facing informatics resources such as The Arabidopsis Information Resource (TAIR), the Bio‐Analytic Resource for Plant Biology (BAR), the Munich Information Centre for Protein Sequences (MIPS), the International Arabidopsis Informatics Consortium (IAIC), and Araport.

The Multinational Arabidopsis Steering Committee (MASC) has represented this global community through its joint oversight of the annual International Conference on Arabidopsis Research (ICAR); production of a MASC annual report^1^; and preparation of a series of decadal roadmaps that have helped coordinate the future activities of the community. These roadmaps were first published in 1990. The first was entitled “A Long‐range Plan for the Genome Research Project”^2^; the second in 2002 was “Beyond the Whole Genome Sequence”^3^; and the third in 2012 was “From Bench to Bountiful Harvests” (Lavagi et al., 2012). The preparation phase for the fourth decadal roadmap has now begun and we encourage input from the Arabidopsis community as we look to 2030 and beyond.

## 1 ARABIDOPSIS RESEARCH REMAINS CUTTING‐EDGE

**FIGURE 1:**
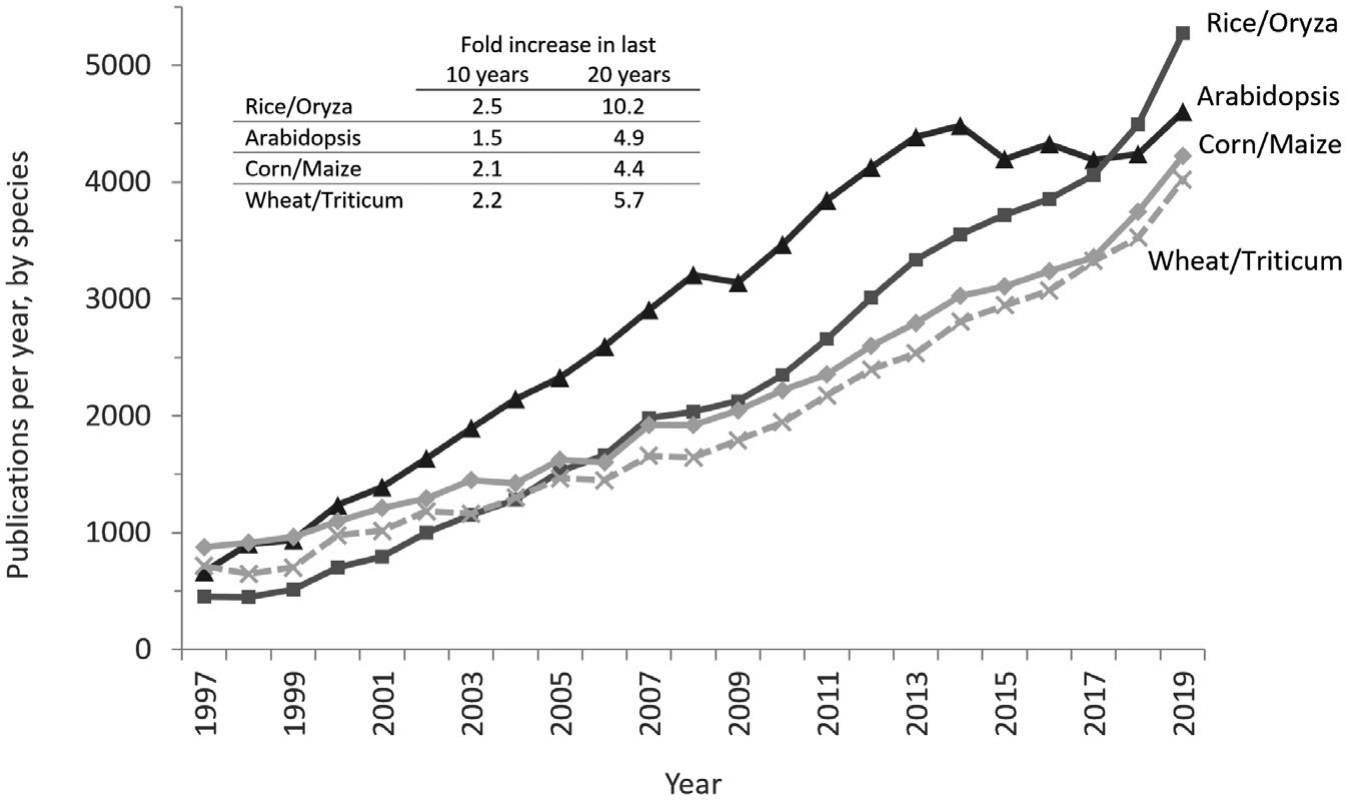
Papers published in PubMed journals globally with Arabidopsis, rice/oryza, corn/maize or wheat/triticum in the Title/Abstract. The following term was used in the PubMed search box: ARABIDOPSIS [Title/Abstract] AND ("journal article"[Publication Type] OR "review"[Publication Type]) AND 2018[DP]

In 2019 according to the U.S. National Center for Biotechnology Information’s PubMed, the annual number of Arabidopsis publications increased after plateauing in 2013 (Figure 1). A significant change occurred in 2018, when, for the first time since we began tracking publication numbers, the number of publications featuring “Rice” or”Oryza” was greater than those featuring Arabidopsis. This might indicate that technological advances in genome sequencing, bioinformatics and gene‐editing, amongst others have recently facilitated research in crop species and that discoveries made in Arabidopsis are now being more effectively translated. This is reinforced by a review by Provart et al. (2016), which surveyed 54,033 Arabidopsis papers and found that in the majority of years, more than 50% of the cited Arabidopsis papers from a given year were referenced by papers in which the research focused on a species other than Arabidopsis. This was determined by the absence of “Arabidopsis” in the taxonomic data available for each paper.

The publication and citation data point to the continued value of Arabidopsis research to plant sciences in general, but more importantly in the area of curiosity‐driven and discovery‐based science. In 2019, many high‐profile “Cell‐Nature‐Science” (CNS) publications featured Arabidopsis research and described several “firsts” in plant science. These included the discovery in plant nuclei of liquid–liquid phase separations of polyadenylation complexes (Fang et al., 2019), a novel mechanism of protein aggregation for controlling auxin responses (Powers et al., 2019), defining the control and organization of the cambial stem cells that are the progenitors of all woody tissues (Miyashima et al., 2019), identifying novel molecular controls of autophagy (Marshall et al., 2019), engineering a synthetic switch to control stomatal opening (Papanatsiou et al., 2019), and defining the first complete blueprint for an immunity pan‐NLRome (Van de Weyer et al., 2019).

This breadth of topics demonstrates that research in Arabidopsis continues to be influential from the perspective of both plant biology and biology more generally. In many cases, the qualities of Arabidopsis that attracted researchers over the past half century (compact size, short generation time, ease of transformation, genomic, and informatic resources) still enable breakthroughs to be made apace. We turn now to efforts to coordinate such resources, and highlight newly generated large datasets that will, in turn, enable further discoveries.

## 2 UPDATE FROM MASC SUBCOMMITTEES

The MASC Subcommittees were established in 2002, at the beginning of the second decadal roadmap, and serve to bring together international groups of scientists who work in a thematic research area. Over the past 18 years these subcommittees have contributed to the MASC annual report, led sessions at ICAR meetings and engaged with the wider community. There are currently eight MASC subcommittees; changes for 2019‐2020 include a new “*Plant Immunity”* subcommittee and the activities of the “*Phenotyping”* subcommittee being subsumed within the International Phenotyping project reports. The other MASC subcommittees are “*Bioinformatics*,” “*Clone‐based ORFeomics*,” “*Epigenetics and Epigenomes,”* “*Metabolomics*,” “*Natural Variation and Comparative Genomics*,” “*Proteomics*” and “*Systems and Synthetic Biology*.” Below we highlight some important findings from each MASC subcommittee.

### 2.1 Proteomics

The Proteomics subcommittee reports that over the past year a significant development in Arabidopsis research came from a study by Mergner et al. (2020) that quantified the total number of detected Arabidopsis proteins (more than 18,000), their dynamic expression range (up to 6‐fold changes in abundance) and their phosphorylation state (43,000 sites across the proteome). In addition, McWhite et al. (2019) generated a complementary database of stable protein complex organization from 13 plant species, including Arabidopsis. These datasets are exciting resources that are available for further analysis and are being integrated into existing community portals such as Proteomics DB (https://www.proteomicsdb.org) and ATHENA (http://athena.proteomics.wzw.tum.de:5002/master_arabidopsisshiny/).

The Mergner et al. (2020) manuscript includes transcriptome information for each protein product yet it does not represent the entirety of the predicted 29,000 open reading frames (dubbed the Arabidopsis “ORFeome”). The MASC ORFeomics subcommittee continues to monitor the resources that provide access to different sets of Arabidopsis clones, each of which are outlined in the MASC annual report. They propose an international project that would catalogue the ORF clones that correspond to the 6,000 remaining uncharacterized protein‐coding genes. It appears that these gene products were not identified in the Mergner et al. (2020) manuscript, so it remains to be discovered if these genes are indeed protein‐coding and if so, where they are localized and what is their functional significance.

### 2.2 Epigenetics and epigenomics

Arabidopsis has been the workhorse for elucidating mechanistic underpinnings of numerous epigenetic phenomena. These studies have both discovered and emphasized the importance of small RNAs, histone modifications, and DNA methylation during epigenome establishment and maintenance, in detection of self from non‐self, and in responding to various environmental challenges. This research is aided by a new community‐facing Arabidopsis RNA‐seq database, ARS, which contains gene expression data from more than 20,000 publicly available RNA‐seq libraries (http://ipf.sustc.edu.cn/pub/athrna/). This resource will dovetail with a soon‐to‐be released resource of whole genome sodium bisulfite sequencing datasets.

### 2.3 Bioinformatics

Over the past year the Arabidopsis bioinformatics community had to overcome the unfortunate loss of funding for the Araport resource. Fortunately, other community projects have stepped up to maintain some of its important features. This includes the hosting of the JBrowse tool at TAIR (https://bit.ly/2Qhb5xC) and the Thalemine instance hosted by the BAR (https://bar.utoronto.ca/thalemine). The third component of the revamped Araport is a new Arabidopsis‐focused instance of the Genome Context Viewer (https://gcv‐arabidopsis.ncgr.org), developed and maintained by Andrew Farmer and Alan Cleary at the National Center for Genome Resources (Cleary and Farmer, 2018), which enables a dynamic comparison of multiple genomes on the basis of their shared functional elements. The GCV includes two sets of newly assembled genomes from 14 different Arabidopsis ecotypes/accessions.

The Bioinformatics subcommittee highlights two outstanding datasets that focus on different aspects of the plant‐pathogen response (Cao et al., 2019; Laflamme et al., 2020). They also highlight a newly released “eFP‐Seq Browser” that enhances exploration of RNA‐seq through the visualization of read map profiles and summary gene expression levels across two large compendia (Sullivan et al., 2019). These resources help to support the key training recommendation for future plant biologists to obtain the skills to develop new software tools in order to extract value from the vast amount of available Arabidopsis ‘Omic data (Argueso et al., 2019).

### 2.4 Metabolomics

The Arabidopsis metabolome is the best studied of all plant species and is used as an exemplar for studies in crop species. The MASC metabolomics subcommittee continues to support the integration of genomic data from natural populations with ecologically relevant metabolomic data that can reveal how a plant has ultimately adapted to environmental stresses. The subcommittee makes the case that as metabolomic platforms become more cost‐effective, and as sensitive as NGS (Next Generation Sequencing) platforms, that metabolomics be considered an equal partner to sister techniques in order to develop a “full picture of a plant.”

### 2.5 Natural variation and comparative genomics

**FIGURE 2:**
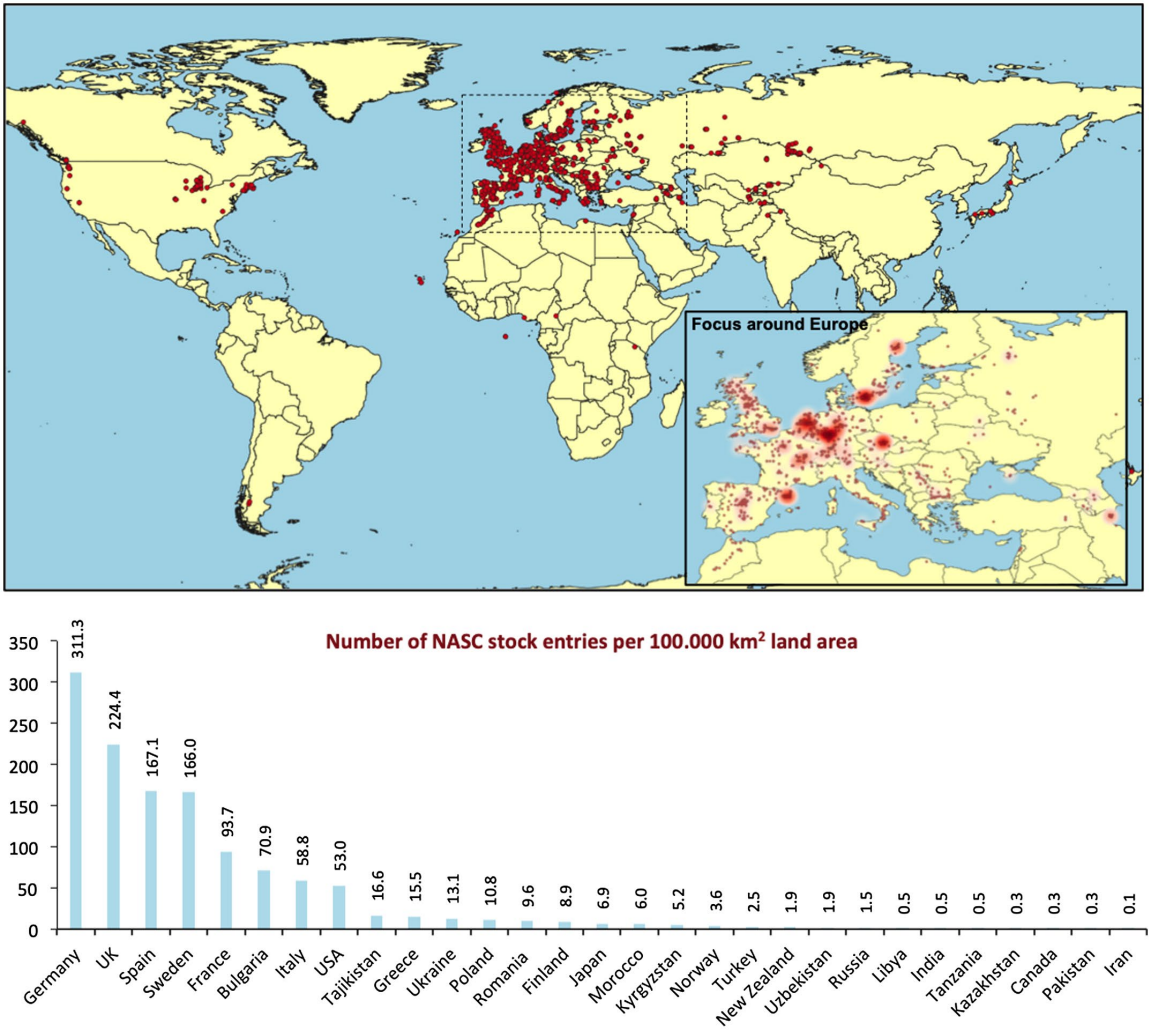
Distribution of NASC natural ecotype collection across the world (Data provided by Marcos Castellanos‐Uribe, Operations Manager at NASC). Focus around Europe shows concentration of the collection on specific regions. Chart below shows number of NASC stock entries per 100.000 km^2^ land area. Note that countries with less than 100.000 km^2^ land area were omitted from the list

During the period of the current decadal roadmap the interrogation of natural variation has been an area of clear success. The hugely successful 1001 Genomes project has led to the development of software tools for further analysis of these publicly available datasets. These including the ViVa: Visualizing Variation (Hamm et al., 2019) and the AraPheno/AraGWAS tools (Togninalli et al., 2020). However, there remains plenty of Arabidopsis geographic variation that has not yet been analyzed. This is highlighted in the MASC country report from Turkey that looks at the distribution of the accessions available for order from the Nottingham Arabidopsis Stock Centre (NASC, Figure 2). This suggests that there are many accessions growing across diverse geographic locations (including Turkey) that are currently underrepresented in the easily accessible available germplasm. In future there are clearly many opportunities for researchers to build upon our understanding of Arabidopsis natural variation through integration of currently underrepresented germplasm.

### 2.6 Systems and synthetic biology

During 2019 there has been significant activity in the research area of the MASC Systems and Synthetic Biology subcommittee, particularly with the development of BAR‐integrated software tool to analyse protein‐protein and protein‐DNA interaction networks (http://bar.utoronto.ca/interactions2/) and in the TuxNet software that analyses RNA‐seq data to infer gene regulatory networks (https://omictools.com/tuxnet‐tool). The subcommittee has been active in the organization of external meetings with the inaugural International Plant Systems Biology Meeting taking place in France in September 2018, with a follow‐up event in Venice postponed until 2021 (https://meetings.embo.org/event/20‐plant‐systems).

### 2.7 Plant immunity

Shahid Mukhtar from University of Alabama at Birmingham is the inaugural chair of the Plant Immunity subcommittee, a necessary initiative that expands MASC activities into an area in which Arabidopsis research has led our current understanding on the molecular mechanisms of pathogen resistance. Over the past year this research area has seen exciting developments that are highlighted within the subcommittee report, which include the first structure of a plant nucleotide‐binding leucine‐rich repeat receptor (NLR; Wang et al., 2019) and a full characterization of the Arabidopsis “NLRome” (Van de Weyer et al., 2019).

## 3 UPDATE ON INTERNATIONAL PROJECTS WITH A FOCUS ON ARABIDOPSIS

A primary example of the collaborative nature of the global Arabidopsis research community is the success of the international stock centres (Nottingham Arabidopsis Stock Centre [NASC], The Arabidopsis Biological Research Center [ABRC] and the RIKEN Bioresource Centre). Despite reporting a downward trend over the past decade, the ABRC distributes annually almost twice as many seed stocks as NASC (190K versus 100K). However, NASC has seen increases over the past few years that undoubtedly reflects that it is the go‐to stock centre for orders from China. The continued success of the stock centres relies on donations of plant material (mostly seeds) from the community and NASC reports that over the past few years German scientists have provided their largest number of donations.

Despite losing government funding over 5 years ago, TAIR continues its excellent biocuration services via an innovative and successful subscription model (Reiser et al., 2016). TAIR has expanded its operations to include collaborations with both PhyloGenes (www.phylogenes.org), a new resource that facilitates inference of gene function based on phylogenetic relationships, and with the open access MicroPublications journal (https://www.micropublication.org/) that facilitates the publication of brief, novel findings, negative and/or reproduced results that may lack a broader scientific narrative. Each week TAIR loads 50–90 papers with the term “Arabidopsis” in the title or abstract into their curation queue. This includes a steady number of papers that report on new functions for previously characterized genes and an increase in the number of papers that describe high‐throughput experiments and contain large datasets. For a variety of reasons, curating from some papers can be challenging so TAIR have produced a document to advise researchers how to make the details of their research more “findable” (https://conf.arabidopsis.org/pages/viewpage.action?pageId=22807345).

Oversight of the Arabidopsis informatics strategy has largely fallen to the International Arabidopsis Informatics Consortium (IAIC), which was funded by the NSF until 2020. In 2018 IAIC hosted a workshop in St Louis and its “take home” recommendation was for the establishment of a centralized “annotation authority” to advise on submissions from groups for new gene names across the Arabidopsis pangenome, to establish a consistent naming scheme, to distribute this format regularly and frequently, and to encourage its adoption (International Arabidopsis Informatics Consortium, 2019). This article also recommends community‐established guidelines and standards for data and metadata formats alongside a searchable, central repository for analysis and visualization tools (such as https://conf.arabidopsis.org/display/COM/Resources). Fortunately, the implementation of these recommendations will be facilitated by a closely linked international community and will undoubtedly be a topic discussed for inclusion within the next roadmap.

The BAR resource continues to be a central hub for researchers to interrogate and visualize their expression data. In addition to its expansion to include data from many other plant species, the BAR is far more than just an “eFP Browser.” The BAR website includes access to a broad set of genomic tools and widgets that have a focus on the analysis of Arabidopsis datasets. The BAR has obtained funding from Genome Canada that will allow the development of a custom “eFP” view in ePlant for a researcher’s own RNA‐seq data as well as the initiation of several new ePlants.

The International Plant Phenotyping community provides excellent links between Arabidopsis research and that conducted in other plant species. The MASC annual report includes brief updates from the International Plant Phenotyping Network (IPPN), European Plant Phenotyping Network (EPPN) and European Infrastructure for Plant Phenotyping (EMPHASIS). An important general feature of these networks is that they host regular calls for applications from researchers to access phenotyping infrastructures where they do not have them available at their home institutions. The European Infrastructure for Plant Phenotyping (EMPHASIS) project is arguably the most interesting development in this area. When it becomes fully operational in 2022 EMPHASIS will place plant phenotyping in a position to obtain centralized European funding through similar mechanisms to those that support other large European infrastructure projects such as the Square Kilometre Array for radio astronomy (although plant phenotyping is *much* cheaper, http://roadmap2018.esfri.eu/).

## 4 MASC COUNTRY UPDATES

The MASC Country Reports provide an overview on the progress of Arabidopsis research on a national scale, cataloguing important publications, new software tools and community resources. Currently, 34 countries with a MASC representative are asked to submit an annual report and 29 of these were able to submit reports in 2020, notwithstanding shutdowns related to the COVID‐19 pandemic. We include some country highlights here.

China is an interesting case study as its country report states that “Arabidopsis is the model plant of choice to many groups. However, only a small portion of these labs is solely dedicated to Arabidopsis research or using [it] as the main model plant…. A major reason behind [this] would be the current funding priority. Whereas there are dedicated grants to basic and applied research in maize, rice, wheat, and virtually each every minor crop, there are no such funding programs towards Arabidopsis research.”

**FIGURE 3:**
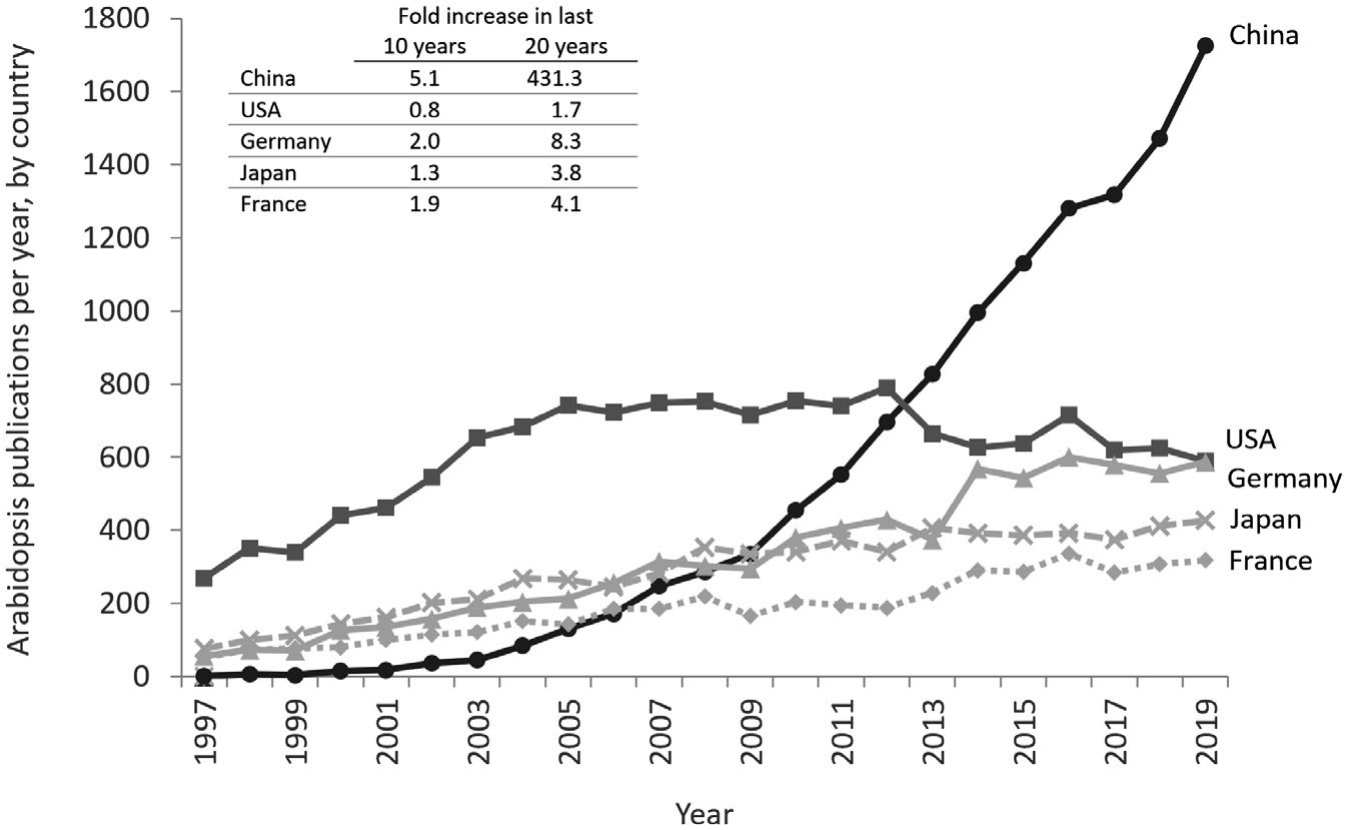
Papers published in PubMed journals with Arabidopsis in the Title/Abstract since 1997. Globally these countries have the highest number of publications in 2019. The following term was used in the PubMed search box: Arabidopsis[Title/Abstract] AND *COUNTRY* AND ("journal article"[Publication Type] OR "review"[Publication Type]) AND *YEAR*[DP]

Although there is somewhat of a plateau in the number of global Arabidopsis publications over the past 5 years, this is not an even trend across all countries (Figures 1 and 3). While numbers of publications on Arabidopsis are steady or falling in some countries, this is offset by the increase in publications coming from Chinese labs, which shows no slow‐down since trending upward a decade ago. This indicates that Chinese researchers are pragmatically continuing to leverage Arabidopsis as a model plant despite a lack of dedicated funding for Arabidopsis research *per se*.

MASC Country representatives provide an opinion on the current status of Arabidopsis research in their countries. It is challenging to obtain a consistent metric for these evaluations as different countries will view their situation from different starting positions. For example, the United Kingdom publishes around 200 Arabidopsis papers per year and there is ~£8M in annual 'Responsive mode' Government funding for “Arabidopsis research” yet this represents a reduction in funding, so the situation is not as healthy as in previous years. However, other countries have low to no funding in research specifically dedicated to Arabidopsis, yet have a positive opinion toward research in this area, for example, “In Brazil even though there is virtually no such funding programs towards Arabidopsis, the number of institutions using Arabidopsis in their research is growing each year. We are seeing a gradual increase in the usage of Arabidopsis as a model plant for molecular and genetic studies due to its power as an easily manipulated model system to investigate gene functions.” Similarly in India, although there is no earmarked financial support for Arabidopsis research from the Government, scientists can compete for grants dedicated for basic science and many projects are regularly funded on Arabidopsis exclusively; “many more projects use Arabidopsis as a system to validate genes from crop plants. Consequently, the overall quality of publications….…has improved considerably.”

Therefore, despite a general admission that there is movement toward more applied research, it is encouraging that many country representatives are positive about the state of Arabidopsis research in their home country. This is exemplified by a response from Belgium; *“*….plant scientists feel an increasing pressure from funding agencies, universities, and research institutes to focus on more applied research aspects. This being said, it is likely that Arabidopsis will remain a major tool to generate and test hypothesis even in applied research projects.” Globally, Arabidopsis clearly remains a critical experimental model for understanding “how plants work,” which will lead to technological advances and knowledge increases that feed into applied projects in a variety of crop plants.

## 5 MOVING TOWARD THE FOURTH ROADMAP

The following broad recommendations were included in the third decadal roadmap (Lavagi et al., 2012):

- Build a predictive model of an Arabidopsis plant from its molecular parts
- Build the International Arabidopsis Informatics Consortium, an international informatics and data infrastructure
- Exploit the wealth of natural variation that exists in Arabidopsis to further our understanding of adaptation and evolution
- Establish an effective knowledge exchange pipeline from the laboratory to the field and vice versa
- Deepen international cooperation and coordination

It is gratifying that progress has been made in each of these areas, yet work remains to be done. At the end of this period we now understand much more about the molecular and biochemical events that control how a plant grows and senses its environment. However, there remain significant gaps in our knowledge, including a lack of understanding of the complex linkages between available ‘omic data sets or the more simple knowledge of how plants sense many of their required nutrients. Improvements in this area are needed to build a fully predictive model that is accurate across time. Fortunately, there is an acknowledgment that bioinformatics training and the development of digital infrastructures are key for the future in depth analysis of Arabidopsis‐derived datasets.

The loss of funding for Araport was disappointing for the community and, although its key activities have been picked up by TAIR, the BAR and the Genome Context Viewer, efforts are needed to ensure that community resources have longevity. This requires the integration of international infrastructures, particularly between Western and Eastern hemispheres. In some areas international cooperation is excellent, such as in the coordination of conference planning, yet elsewhere it can be improved. These challenges include, but are not limited to, implementation of effective mechanisms of data sharing, cultural and language differences, and availability of global funding initiatives.

With this update, MASC calls on all Arabidopsis researchers to consider areas for inclusion in the next decadal roadmap. We expect participation from long‐time community leaders, such as the North American Arabidopsis Steering Committee (NAASC), and collaborators from the United States, Germany, and Japan. We also expect that there will be contributions toward decadal priorities from a broader group of MASC members, especially those representing countries with significant and increasing Arabidopsis research, such as China or India. The positive sense of East‐West collaboration that was felt by those who attended ICAR2019 in Wuhan was a promising beginning to these discussions.

## 6 POSSIBLE AREAS FOR INCLUSION IN THE NEXT DECADAL ROADMAP

- What are the strategies that might be used to build globally sustainable digital infrastructures to support the integration of multi‐omic datasets?
- How can both the data and metadata from complex multi‐omic experiments be collated and shared for the benefit of the wider community in order to feed into translational pipelines?
- How can we integrate mechanistic and quantitative genetic insights to enable plant acclimation to vastly different climates, within a very short time period?
- How can the community build internationally cohesive and diverse collaborative teams of scientists to answer important questions in plant science?

Over the next year, these ideas will be developed and will coalesce during discussions at a MASC‐supported discussion session at ICAR2021 in Seattle. The roadmap will be launched and published prior to ICAR2022 in Belfast and will hopefully lead in the planning of community‐driven projects over the coming decade.

## AUTHOR CONTRIBUTIONS

GP and NP conceived the manuscript, GP, NP, SB, and BU contributed to the manuscript. Members of Multinational Arabidopsis Steering Committee (MASC) provided background content.

## ENDNOTES

^1^
http://arabidopsisresearch.org/index.php/en/publications

^2^
http://arabidopsisresearch.org/images/publications/mascreports/1990_MASCPlan.pdf

^3^
http://arabidopsisresearch.org/images/publications/mascreports/2002_MASCreport.pdf

## Appendix 1

### Multinational Arabidopsis Steering Committee

Keith Adams, University of British Columbia, Canada; Wagner Araújo, Universidade Federal de Viçosa, Brazil; Sébastien Aubourg, INRA, France; Sacha Baginsky, Martin‐Luther‐Universität Halle‐Wittenberg, Germany; Erica Bakker, TAIR, USA; Katja Bärenfaller, Federal Institute of Technology, Switzerland; Jacqui Batley, University of Western Australia, Australia; Mike Beale, Rothamsted Research, UK; Mark Beilstein, University of Arizona, USA; Youssef Belkhadir, Gregor Mendel Institute of Molecular Plant Biology, Austria; Tanya Berardini, TAIR, USA; Joy Bergelson, University of Chicago, USA; Francisca Blanco‐Herrera, Universidad Andres Bello, Chile.; Siobhan Brady, University of California Davis, USA; Hans‐Peter Braun, Leibniz Universität Hannover, Germany; Steve Briggs, University of California, USA; Lynette Brownfield, University of Otago, New Zealand; Maura Cardarelli, IBPM‐National Research Council, c/o Sapienza University of Rome; Marcos Castellanos‐Uribe, NASC Operations Manager, University of Nottingham, UK; Gloria Coruzzi, NYU Center for Genomics & Systems Biology, USA; Maheshi Dassanayake, Louisiana State University, USA; Geert De Jaeger, VIB UGhent, Belgium; Brian Dilkes, Purdue University, USA; Colleen Doherty, North Carolina State University, USA; Joe Ecker, Salk Institute, USA; Pat Edger, Michigan State University, USA; David Edwards, University of Western Australia, Australia; Farid El Kasmi, University of Tuebingen, Germany; Maria Eriksson, Umeå University, Sweden; Moises Exposito‐Alonso, Carnegie Institution for Science and Stanford University, California, USA; Pascal Falter‐Braun, Helmholtz Zentrum München, Germany; Alisdair Fernie, Max Planck Institute for Molecular Plant Physiology, Germany; Myriam Ferro, CEA, Grenoble, France; Oliver Fiehn, University of California, USA; Joanna Friesner, IAIC Assistant, University of California Davis, USA; Katie Greenham, University of Minnesota, USA; Yalong Guo, Institute of Botany, Chinese Academy of Sciences, China; Thorsten Hamann, Norwegian University of Science and Technology, Norway; Angela Hancock, MPI‐Cologne, Germany; Marie‐Theres Hauser, BOKU Vienna, Austria; Joshua Heazlewood, University of Melbourne, Australia; Cheng‐Hsun Ho, Academia Sinica, Taiwan; Hanna Hõrak, University of Tartu, Estonia; Eva Huala, TAIR, USA; Inhwan Hwang, Pohang University of Science and Technology, South Korea; Satoshi Iuchi, RIKEN BRC, Japan; Pankaj Jaiswal, Oregon State University, USA; Liina Jakobson, Estonian Crop Research Institute, Estonia; Yunhe Jiang, KAUST, Saudi Arabia; Yuling Jiao, Institute of Genetics and Developmental Biology, China; Alexandra Jones, University of Warwick, UK; Yasuhiro Kadota, RIKEN CSRS, Japan; Jitendra Khurana, University of Delhi, India; Dan Kliebenstein, University of California Davis, USA; Emma Knee, ABRC Associate Director, Ohio State University, USA; Masatomo Kobayashi, RIKEN BRC, Japan; Marcus Koch, Heidelberg University, Germany; Gabriel Krouk, CNRS, France; Tony Larson, University of York, UK; Rob Last, Michigan State University, USA; Loïc Lepiniec, IJPB, INRAE Versailles, Université Paris‐Saclay, France; Song Li, Virginia Tech University, USA; Claire Lurin, Institute of Plant Sciences ‐Paris‐ Saclay, France; Martin Lysak, CEITEC, Czech Republic; Steven Maere, VIB UGhent University, Belgium; Robert Malinowski, Department of Integrative Plant Biology Institute of Plant Genetics of the Polish Academy of Sciences, Poznan, POLAND; Florian Maumus, INRA, Versailles, France; Sean May, NASC Director, University of Nottingham, UK; Klaus Mayer, Helmholtz Zentrum München, Germany; David Mendoza‐Cozatl, University of Missouri, USA; Isabel Mendoza‐Poudereux, Global Plant Council, Spain; Blake Meyers, IAIC Director, The Donald Danforth Plant Science Center and the University of Missouri ‐ Columbia; José Luis Micol, Universidad Miguel Hernández, Spain; Harvey Millar, University of Western Australia, Australia; Hans‐Peter Mock, Institute for Plant Genetics and Crop Plant Research, Germany; Karolina Mukhtar, University of Alabama at Birmingham, USA; Shahid Mukhtar, University of Alabama at Birmingham, USA; Monika Murcha, The University of Western Australia, Australia; Hirofumi Nakagami, RIKEN CSRS, Japan; Yasukazu Nakamura, Kazusa DNA Research Institute, Japan; Luke Nicolov, UCLA, USA; Basil Nikolau, Iowa State University, USA; Moritz Nowack, VIB‐Ugent, Belgium; Adriano Nunes‐Nesi, Universidade Federal de Viçosa, Brazil; Michael Palmgren, University of Copenhagen, Denmark; Geraint Parry, Cardiff University, UK; Nicola Patron, Earlham Institute, UK; Scott Peck, University of Missouri, USA; Ullas Pedmale, CSHL, USA; Catherine Perrot‐Rechenmann, Institut Jean‐Pierre Bourgin (IJPB) Versailles, France; Roland Pieruschka, Julich Plant Phenotyping Centre, Germany; José Pío‐Beltrán, IBMCP‐CSIC, Spain; J. Chris Pires, University of Missouri, USA; Nicholas Provart, University of Toronto, Canada; Loïc Rajjou, AgroParisTech, France; Leonore Reiser, TAIR, USA; Sigrun Reumann, University of Stavanger, Norway; Sue Rhee, Carnegie Institution for Science and Stanford University, California, USA; Stamatis Rigas, Agricultural University of Athens, Greece; Norbert Rolland, CEA, Grenoble, France; Andres Romanowski, Fundación Instituto Leloir, Argentina; Véronique Santoni, INRA, France; Sigal Savaldi‐Goldstein, Technion––Israel Institute of Technology, Israel; Robert Schmitz, University of Georgia, USA; Waltraud Schulze, University of Hohenheim, Germany; Motoaki Seki, RIKEN CSRS, Japan; Kentaro K. Shimizu, University of Zurich, Switzerland; Keith Slotkin, Donald Danforth Center, USA; Ian Small, University of Western Australia, Australia; David Somers, ABRC Director, Ohio State University, USA; Rosangela Sozzani, North Carolina State University, USA; Charles Spillane, National University of Ireland Galway, Ireland; Ramamurthy Srinivasan, National Institute for Plant Biology, IARI, New Delhi and Sharda University, India; Nicolas Taylor, The University of Western Australia, Australia; Marcela‐Karey Tello‐Ruiz, CSHL, USA; Jay Thelen, University of Missouri, USA; Takayuki Tohge, RIKEN CSRS, Japan; Christopher Town, J. Craig Venter Institute, USA; Tetsuro Toyoda, RIKEN BASE, Japan; Baris Uzilday, Department of Biology, Faculty of Science, Ege University, Izmir, Turkey; Yves Van De Peer, VIB/ UGent, Belgium; Klaas van Wijk, Cornell University, USA; Philipp von Gillhaussen, Julich Plant Phenotyping Centre, Germany; Justin Walley, Iowa State University, USA; Doreen Ware, CSHL, USA; Wolfram Weckwerth, University of Vienna, Austria; Julian Whitelegge, University of California, USA; Stefanie Wienkoop, University of Vienna, Austria; Clay Wright, Virginia Tech University, USA; Michael Wrzaczek, University of Helsinki, Helsinki, Finland; Misako Yamazaki, University of Zurich, Switzerland; Marcelo Yanovsky, Fundación Instituto Leloir, Argentina; Viktor Žárský, Charles University, Czech Republic; Xuehua Zhong, University of Wisconsin‐Madison, USA.
